# Neuronal ApoE Regulates the Cell-to-Cell Transmission of α-Synuclein

**DOI:** 10.3390/ijms23158311

**Published:** 2022-07-27

**Authors:** Seo-Jun Kang, Soo-Jeong Kim, Hye Rin Noh, Beom Jin Kim, Jae-Bong Kim, Uram Jin, Sun Ah Park, Sang Myun Park

**Affiliations:** 1 Department of Pharmacology, Ajou University School of Medicine, Suwon 16499, Korea; kkw1118@naver.com (S.-J.K.); gpfls9341@gmail.com (H.R.N.); qjawls3394@naver.com (B.J.K.); gacc2104@naver.com (J.-B.K.); statery@naver.com (U.J.); 2 Center for Convergence Research of Neurological Disorders, Ajou University School of Medicine, Suwon 16499, Korea; isabell88@daum.net (S.-J.K.); sap001@ajou.ac.kr (S.A.P.); 3 Neuroscience Graduate Program, Department of Biomedical Sciences, Ajou University School of Medicine, Suwon 16499, Korea; 4 Department of Cardiology, Ajou University School of Medicine, Suwon 16499, Korea; 5 Department of Anatomy, Ajou University School of Medicine, Suwon 16499, Korea; 6 Department of Neurology, Ajou University School of Medicine, Suwon 16499, Korea

**Keywords:** Parkinson’s disease, Lewy body, α-synuclein propagation, α-synuclein uptake, α-synuclein release, ApoE

## Abstract

The presence of protein inclusions, called Lewy bodies (LBs) and Lewy neurites (LNs), in the brain is the main feature of Parkinson’s disease (PD). Recent evidence that the prion-like propagation of α-synuclein (α-syn), as a major component of LBs and LNs, plays an important role in the progression of PD has gained much attention, although the molecular mechanism remains unclear. In this study, we evaluated whether neuronal ApoE regulates the cell-to-cell transmission of α-syn and explored its molecular mechanism using in vitro and in vivo model systems. We demonstrate that neuronal ApoE deficiency attenuates both α-syn uptake and release by downregulating LRP-1 and LDLR expression and enhancing chaperone-mediated autophagy activity, respectively, thereby contributing to α-syn propagation. In addition, we observed that α-syn propagation was attenuated in ApoE knockout mice injected with pre-formed mouse α-syn fibrils. This study will help our understanding of the molecular mechanisms underlying α-syn propagation.

## 1. Introduction

Parkinson’s disease (PD) is characterized by two main pathological features: the degeneration of midbrain dopaminergic neurons and the presence of intraneuronal inclusions called Lewy bodies (LBs) and Lewy neurites (LNs). LBs and LNs are mainly composed of α-synuclein (α-syn) aggregates [1]. Mutations in the α-syn gene have been found in early onset familial PD, and genome-wide association studies revealed a strong association between the α-syn gene and sporadic PD [2,3,4]. In addition to PD, the accumulation of insoluble α-syn aggregates has also been observed in other neurodegenerative disorders, such as dementia with Lewy bodies and multiple system atrophy, which are referred to as α-synucleinopathies, suggesting that α-syn plays an important role in their pathogenesis [5]. Recently, the prion-like propagation of α-syn has been reported to play an important role in the progression of PD [6,7,8]. In this process, α-syn aggregates are released from cells and taken up by neighboring cells; however, the molecular mechanism underlying each process remains unknown.

ApoE is the predominant apolipoprotein in the central nervous system (CNS) and acts as a scaffold for the formation of high-density lipoprotein (HDL)-like particles found in the CNS [9]. Human ApoE is primarily expressed in three isoforms (ApoE2, ApoE3, and ApoE4) that differ by only two residues. Specifically, ApoE4 strongly increases the risk of developing late-onset Alzheimer’s disease (AD) [10,11,12], whereas ApoE2 reduces the risk of AD relative to ApoE3 [13]. In addition, ApoE expression is associated with AD [14]. Although the evidence is not as strong as that for AD, ApoE isoforms have been reported to be associated with PD. The age at onset for ApoE4 is significantly lower than that for ApoE3 and ApoE2 in PD [15]. ApoE4 induces an increase in the number of cortical LBs and amyloid plaques in PD [16]. In contrast, ApoE2, but not ApoE4, has been reported to be positively associated with sporadic PD [17]. Furthermore, ApoE has been reported to be increased in neurons and LBs of patients with PD [18,19]. An increased level of ApoE in the cerebrospinal fluid (CSF) of patients with early PD has also been reported [19]. In α-syn transgenic mice, ApoE levels are increased and the deletion of ApoE delays the neurodegeneration caused by α-syn [20]. ApoE4 has also been reported to exacerbate α-syn pathology in human ApoE knock-in (KI) animal models [21,22] and α-syn seeding activity and neurotoxicity in AD [23].

ApoE is mainly produced in astrocytes [24] and delivers cholesterol and other lipids to neurons through ApoE receptors, such as low-density lipoprotein receptor (LDLR) and low-density lipoprotein receptor-related protein 1 (LRP-1) [25]. However, ApoE is known to be produced in neurons when a specific stress condition such as oxidative stress or excitotoxic damage occurs [26,27,28,29], and neuronal ApoE has been reported to regulate synaptic plasticity as well as learning and memory [30,31,32]. Nevertheless, the role of neuronal ApoE has not been extensively studied. In this study, we investigate whether neuronal ApoE regulates the cell-to-cell transmission of α-syn and explore the underlying molecular mechanism.

## 2. Results

### 2.1. Neuronal ApoE Regulates the Uptake of α-Syn into Neurons

**Figure 1:**
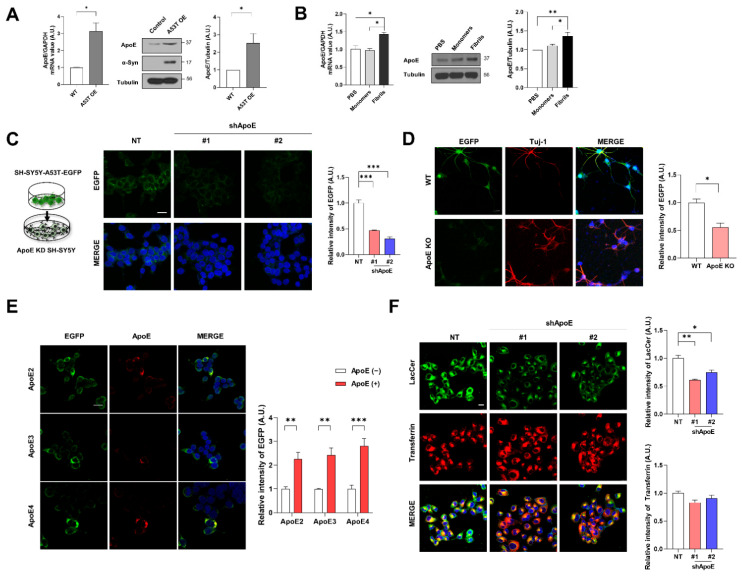
ApoE deficiency inhibits α-syn uptake into neurons. (**A**) ApoE expression in SH-SY5Y and A53T-α-syn OE SH-SY5Y cells was analyzed by RT-PCR and Western blot. Values were derived from three independent experiments (*n* = 3). (**B**) After treatment with 1 μM α-syn monomers and fibrils for 24 h, the cells were analyzed by RT-PCR and Western blot. Values were derived from three independent experiments (*n* = 3). (C, D) NT, ApoE KD #1 and #2 SH-SY5Y cells (**C**) and primary neurons (**D**) were co-cultured with differentiated A53T α-syn-EGFP OE SH-SY5Y cells for 24 h using a dual chamber. The cells were then fixed and immunostained with anti-EGFP or anti-Tuj-1 antibodies. Values were derived from three independent experiments (*n* = 3). (**E**) SH-SY5Y cells were transiently transfected with plasmids of ApoE isoforms (ApoE2, ApoE3, or ApoE4). The cells were co-cultured with differentiated A53T-α-syn-EGFP OE SH-SY5Y cells using a dual chamber. The cells were then fixed and immunostained with anti-EGFP and anti-ApoE antibodies. Values were derived from three independent experiments (*n* = 3). (**F**) NT, ApoE KD #1, and #2 SH-SY5Y cells were incubated with 50 nM BOIPY^®^ FL C5-Lactosylceramide and 2.5 μg/mL rhodamine-conjugated transferrin for 60 min. The intensity was analyzed with ImageJ and the LASX program. Values were derived from three independent experiments. * *p* < 0.05, ** *p* < 0.01, *** *p* < 0.001 against control; one-way ANOVA or unpaired t-tests. Blue indicates DAPI staining. Scale bars indicate 20 μm.

To explore whether ApoE is associated with α-syn pathology, we first examined ApoE expression in SH-SY5Y cells overexpressing (OE) A53T α-syn. As shown in Figure 1A, we observed that ApoE mRNA and protein levels were increased in A53T α-syn OE SH-SY5Y cells compared with the control. The expression of ApoE was also increased by treatment with α-syn fibrils, but not α-syn monomers (Figure 1B), suggesting that α-syn affects ApoE expression. Next, we generated ApoE knockdown (KD) SH-SY5Y stable cell lines. The expression of ApoE in both cell lines was efficiently decreased, while α-syn expression was not affected (Figure S1A,B). To explore whether neuronal ApoE regulates the uptake of α-syn, we performed a dual-chamber assay in which the uptake of cell-derived A53T α-syn-EGFP into cells was monitored [33]. A53T α-syn-EGFP was taken up to a lesser extent by ApoE KD SH-SY5Y cells (Figure 1C). Similar results were seen in ApoE knockout (KO) primary neurons (Figure 1D). The overexpression of ApoE isoforms (ApoE2, ApoE3, and ApoE4) in SH-SY5Y cells enhanced the uptake of α-syn. However, there were no differences among ApoE isoforms (Figure 1E), suggesting that neuronal ApoE regulates the uptake of α-syn irrespective of the ApoE isoform. To investigate whether ApoE regulates the uptake of α-syn specifically, an in vitro endocytosis assay using transferrin and lactosylceramide (LacCer) as a marker for clathrin-dependent and lipid raft-dependent endocytosis, respectively, was performed [34,35]. As shown in Figure 1F, lipid raft-dependent endocytosis decreased in ApoE KD SH-SY5Y cells, whereas clathrin-dependent endocytosis did not change, suggesting that ApoE regulates lipid raft-dependent endocytosis.

### 2.2. ApoE Deficiency Increases Intracellular Cholesterol Levels and Decreases Apoe Receptor Expression in Neurons

**Figure 2:**
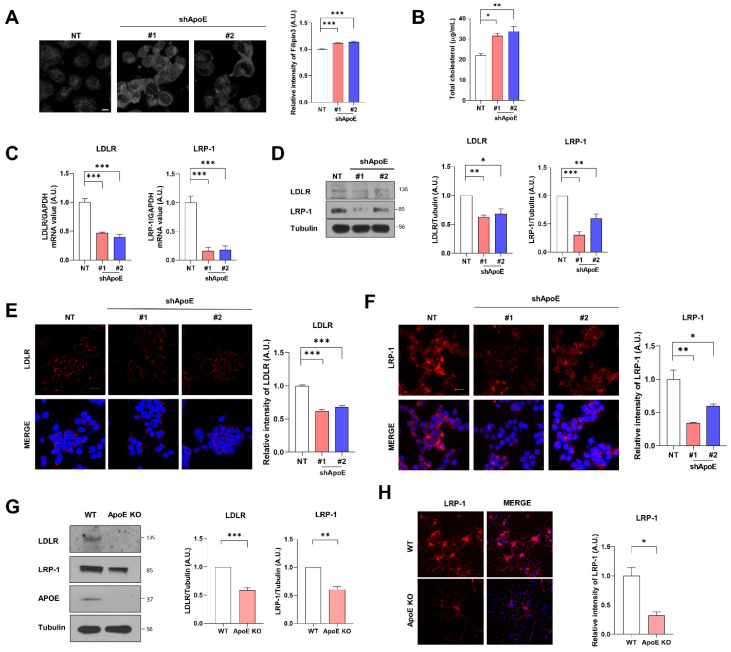
ApoE deficiency increases intracellular cholesterol levels and decreases the expression of ApoE receptors in neurons. (**A**) The cells were fixed and stained with filipin3. Values were derived from three independent experiments (*n* = 3). (**B**) Total cholesterol levels were measured with a cholesterol assay kit. Values were derived from three independent experiments (*n* = 3). (**C**–**F**) LDLR and LRP-1 levels were measured with RT-PCR (**C**), Western blot (**D**), and immunostaining with anti-LDLR (E) and anti-LRP-1 (**F**) antibodies. Values were derived from three independent experiments (*n* = 3). (**G**) Primary neurons from WT and ApoE KO mice were lysed with RIPA buffer, and then the samples were analyzed by Western blot. Values were derived from three independent experiments (*n* = 3). (**H**) Primary neurons from WT and ApoE KO mice were fixed and immunostained with anti-LRP-1. Values were derived from three independent experiments (*n* = 3). * *p* < 0.05, ** *p* < 0.01, *** *p* < 0.001 against control; one-way ANOVA or unpaired t-tests. Blue indicates DAPI staining. Scale bars indicate 20 μm.

Cholesterol homeostasis is regulated by ApoE in cells through cholesterol transfer [36,37]. We measured intracellular cholesterol levels to investigate the role of neuronal ApoE in cellular cholesterol levels. Immunocytochemistry with filipin3, which stains cholesterol in cells [38,39], showed that intracellular cholesterol levels were increased in ApoE KD SH-SY5Y cells than in the control cells (Figure 2A). The total cellular cholesterol levels measured with ELISA showed a similar pattern (Figure 2B), suggesting that ApoE deficiency increases intracellular cholesterol levels in neurons. Intracellular cholesterol regulates the expression of ApoE receptors [40]. The mRNA and protein levels of LDLR and LRP-1 decreased in ApoE KD SH-SY5Y cells (Figure 2C,D). Immunocytochemistry for LDLR and LRP-1 showed similar results (Figure 2E,F). In primary neurons from ApoE KO mice, LDLR and LRP-1 levels were also decreased (Figure 2G,H), suggesting that ApoE KD increases intracellular cholesterol levels and further decreases ApoE receptors, such as LDLR and LRP-1, in neurons.

### 2.3. LDLR and LRP-1 Expressed in SH-SY5Y Cells Function as Receptors Responsible for α-Syn Uptake

**Figure 3:**
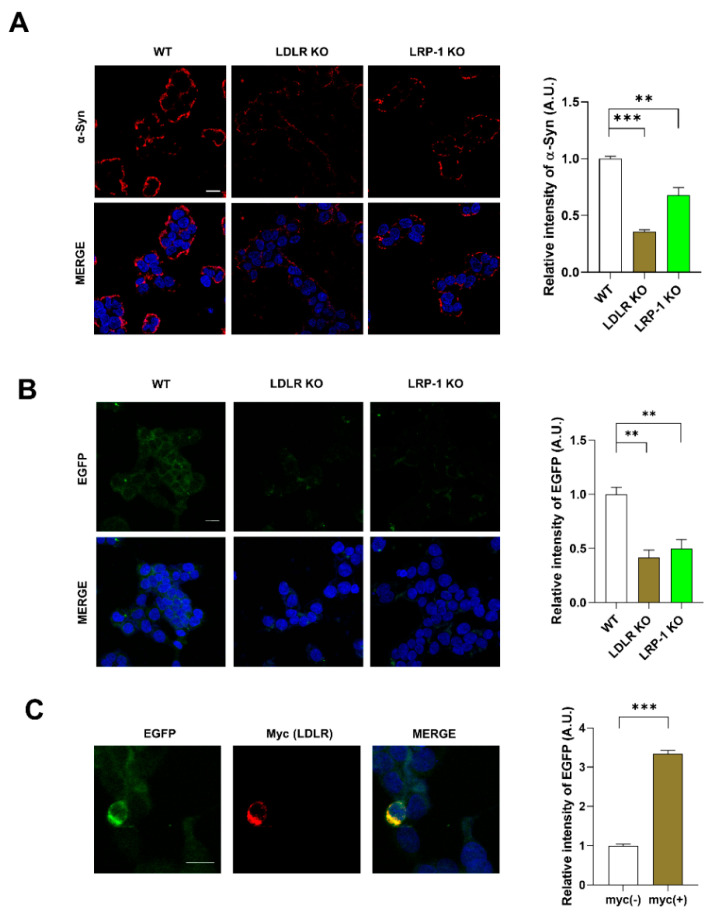
LDLR and LRP-1 expressed in SH-SY5Y cells function as receptors responsible for α-syn uptake. (**A**) LDLR KO and LRP-1 KO SH-SY5Y cells were treated with 1 μM α-syn fibrils for 30 min, and the cells were then fixed and immunostained with an anti-α-syn antibody. Values were derived from three independent experiments (*n* = 3). (**B**) LDLR KO and LRP-1 KO SH-SY5Y cells were co-cultured with differentiated A53T α-syn-EGFP OE SH-SY5Y cells for 24 h using a dual chamber. The cells were then fixed and immunostained with an anti-EGFP antibody. Values were derived from three independent experiments (*n* = 3) (**C**) SH-SY5Y cells were transfected with a plasmid for LDLR-myc. The cells were co-cultured with differentiated A53T α-syn-EGFP OE SH-SY5Y cells for 24 h using a dual chamber. The cells were then fixed and immunostained with anti-Myc and anti-EGFP antibodies. Values were derived from three independent experiments (*n* = 3). ** *p* < 0.01, *** *p* < 0.001 against the control; one-way ANOVA or unpaired t-tests. Blue indicates DAPI staining. Scale bars indicate 20 μm.

Cells partially take up α-syn by receptor-mediated endocytosis [8]. To explore the possibility that LDLR or LRP-1 function as receptors for α-syn uptake, we generated LDLR and LRP-1 KO SH-SY5Y cells (Figure S1C). We observed that the interaction of α-syn fibrils with the plasma membrane of LDLR and LRP-1 KO cells was decreased (Figure 3A), suggesting that LDLR and LRP-1 both may function as receptors for α-syn fibrils. Next, we explored whether LDLR and LRP-1, expressed in neurons, mediate α-syn uptake. The dual chamber assay showed that less cell-derived A53T α-syn-EGFP was taken up by LDLR and LRP-1 KO SH-SY5Y cells, similar to what we observed for ApoE KD cells, compared with the control (Figure 3B). In contrast, LDLR overexpression increased α-syn uptake (Figure 3C), suggesting that LDLR and LRP-1 mediate α-syn uptake.

### 2.4. ApoE Deficiency in SH-SY5Y Cells Inhibits α-Syn Propagation

**Figure 4:**
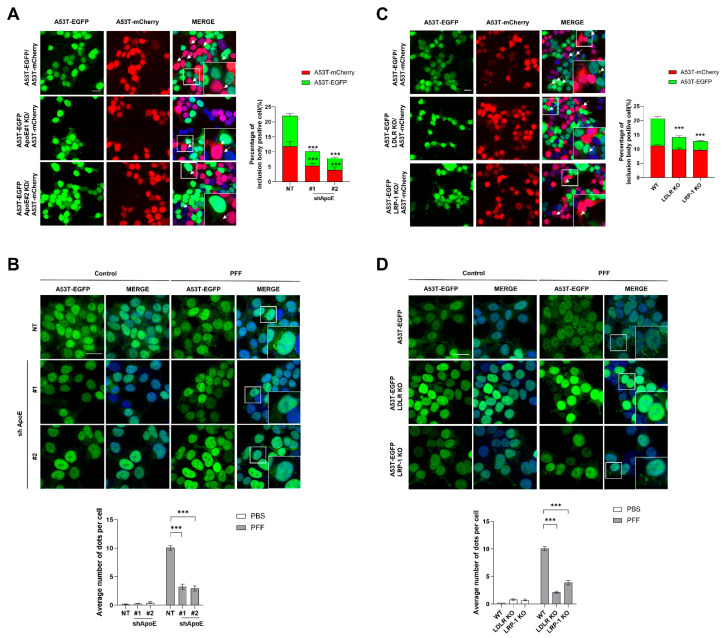
ApoE deficiency inhibits α-syn propagation. (**A**) NT and ApoE KD #1, #2/A53T α-syn-EGFP OE SH-SY5Y cells were cocultured with A53T α-syn-mCherry OE SH-SY5Y cells for 5 days in the presence of 50 μM RA. The samples were then observed under a confocal microscope, and the number of cells containing double fluorescence-labeled puncta was analyzed. Values were derived from three independent experiments (*n* = 3). (**B**) After the treatment of NT and ApoE KD #1, #2/A53T α-syn-EGFP OE SH-SY5Y cells with 1 μM α-syn fibrils for 24 h, the cells were fixed, the samples were analyzed by confocal microscopy, and the number of aggregated puncta was counted with MetaMorph software. Nuclear images based on DAPI were precluded. Values were derived from three independent experiments (*n* = 3). (**C**) NT and LDLR KO, LRP-1 KO/A53T α-syn-EGFP OE SH-SY5Y cells were cocultured with A53T α-syn-mCherry OE SH-SY5Y cells for 5 days in the presence of 50 μM RA. Then, the samples were observed under a confocal microscope, and the number of cells containing double fluorescence-labeled puncta was analyzed. Values were derived from three independent experiments (*n* = 3). (**D**) After the treatment of NT and LDLR KO, LRP-1 KO/A53T α-syn-EGFP OE SH-SY5Y cells with 1 μM α-syn fibril for 24 h, the cells were fixed. The samples were then analyzed by confocal microscopy and the number of aggregated puncta was counted with MetaMorph software. Values were derived from three independent experiments (*n* = 3). *** *p* < 0.001 against the control; one-way ANOVA. Blue indicates DAPI staining. Scale bars indicate 20 μm.

To further explore whether neuronal ApoE regulates α-syn propagation, we generated stable cell lines OE A53T α-syn-EGFP in ApoE-KD SH-SY5Y cells (Figure S1D). Then, a coculture assay, described in a previous study that explored whether cell-derived α-syn can induce inclusion body formation with endogenous α-syn expressed in neurons by measuring double fluorescence-labeled aggregation puncta [33], was performed. When ApoE KD/A53T α-syn-EGFP OE SH-SY5Y cells were cocultured with A53T α-syn-mCherry OE SH-SY5Y cells, double fluorescence-labeled aggregation puncta in ApoE KD/A53T α-syn-EGFP OE SH-SY5Y cells were efficiently suppressed. Interestingly, double fluorescence-labeled aggregation puncta in A53T α-syn-mCherry OE SH-SY5Y cells were also suppressed (Figure 4A), suggesting that the release of α-syn from ApoE KD/A53T α-syn-EGFP OE SH-SY5Y cells may also be decreased. Treatment with recombinant α-syn fibrils induces intracellular α-syn aggregation [41,42,43]. When A53T α-syn-EGFP OE SH-SY5Y cells were treated with α-syn fibrils, the aggregation of A53T α-syn-EGFP was observed. In ApoE KD SH-SY5Y cells, fewer aggregation puncta were observed (Figure 4B), which supports the results of the co-culture assay. In LDLR and LRP-1 KO cells, aggregation puncta were efficiently suppressed in LDLR and LRP-1 KO/A53T α-syn-EGFP OE SH-SY5Y cells, but not in A53T α-syn-mCherry OE SH-SY5Y cells (Figure 4C). Fewer aggregation puncta in LDLR and LRP-1 KO/A53T α-syn-EGFP OE SH-SY5Y cells were also observed after the treatment with α-syn fibrils (Figure 4D). These results suggest that ApoE and ApoE receptors may be differentially involved in α-syn propagation in SH-SY5Y cells.

### 2.5. ApoE Deficiency Inhibits α-Syn Release in SH-SY5Y Cells

**Figure 5:**
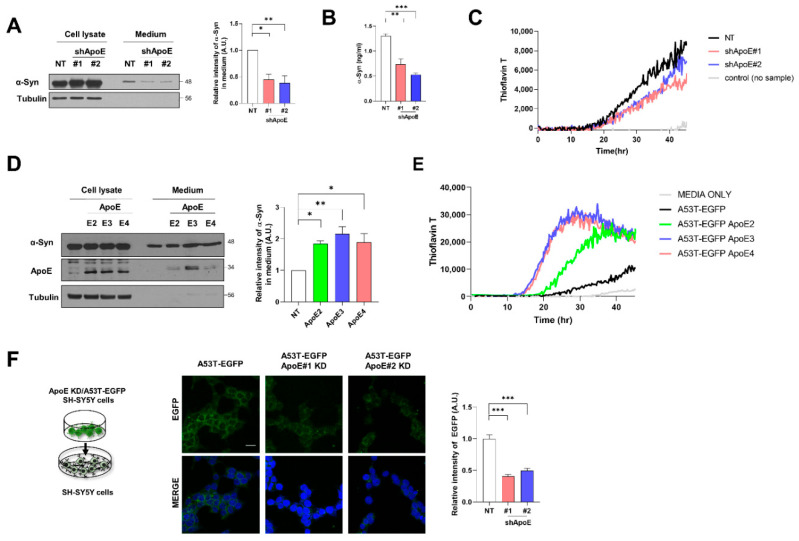
ApoE deficiency inhibits α-syn release in SH-SY5Y cells. (**A**) Differentiated NT and ApoE KD #1, #2/A53T α-syn-EGFP OE SH-SY5Y cells were cultured in serum-free media for 1 day, and culture media were collected. The cells were lysed with RIPA buffer and culture media were 50-fold concentrated. The samples were then analyzed by Western blot. Values were derived from three independent experiments (*n* = 3). (**B**) The α-syn in culture media was analyzed by ELISA (*n* = 3). (**C**) Culture media were analyzed by α-syn RT-QUIC. Values were derived from triplicate and a representative of three independent experiments (*n* = 3). (**D**) A53T α-syn-EGFP OE SH-SY5Y cells were transfected with plasmids for ApoE isoforms (ApoE2, ApoE3, and ApoE4) and differentiated by 50 μM RA for 5 days. The cells were then cultured in serum-free media for 1 day and culture media were collected. Cells were lysed with RIPA buffer and culture media were 50-fold concentrated. The samples were then analyzed by Western blot. Values were derived from three independent experiments (*n* = 3). (**E**) Culture media were analyzed by α-syn RT-QUIC. Values were derived from triplicate and a representative of three independent experiments (*n* = 3). (**F**) SH-SY5Y cells in the lower chamber were cocultured with differentiated NT, ApoE KD #1, or #2/A53T α-syn-EGFP OE SH-SY5Y cells for 24 h. The cells in the lower chamber were immunostained with an anti-EGFP antibody. Values were derived from three independent experiments (*n* = 3). * *p* < 0.05, ** *p* < 0.01, *** *p* < 0.001 against the control; one-way ANOVA. Blue indicates DAPI staining. Scale bar indicates 20 μm.

To explore whether ApoE affects α-syn release from neurons, we measured the released α-syn in the culture media. As shown in Figure 5A, on Western blotting, α-syn was less abundant in the culture media from ApoE KD/A53T α-syn-EGFP OE SH-SY5Y cells than in the media from A53T α-syn-EGFP OE SH-SY5Y cells. This was also confirmed by ELISA (Figure 5B). Furthermore, α-syn is released in both monomeric and aggregated forms [8]. Extracellular α-syn aggregates are responsible for the intracellular aggregation of endogenous α-syn as a seed [44]. Recently, an α-syn real-time quaking-induced conversion (RT-QUIC) analysis was developed to detect α-syn aggregates in various biological samples [45]. We performed such α-syn RT-QUIC analysis to detect α-syn fibrils as seeds in culture media. As shown in Figure 5C, α-syn fibrils were detected in the culture media of both cell lines. However, fewer responses were detected in the culture medium of ApoE KD/A53T α-syn-EGFP OE SH-SY5Y cells. In contrast, the overexpression of ApoE isoforms in A53T α-syn-EGFP SHSY5Y cells induced a greater release of α-syn from cells (Figure 5D). Our α-syn RT-QUIC analysis also showed a greater response in the culture media from A53T α-syn-EGFP SHSY5Y cells OE ApoE isoforms (Figure 5E). Additionally, a dual-chamber assay was performed using ApoE KD/A53T α-syn-EGFP OE SH-SY5Y cells as donor cells and the control SH-SY5Y cells as recipient cells. Reduced levels of A53T α-syn-EGFP were detected in SH-SY5Y cells with ApoE KD/A53T α-syn-EGFP OE SH-SY5Y cells than in A53T α-syn-EGFP OE SH-SY5Y cells in the upper chamber (Figure 5F), suggesting that ApoE also regulates α-syn release from SH-SY5Y cells.

### 2.6. ApoE Deficiency Enhances Chaperone-Mediated Autophagy in SH-SY5Y Cells

**Figure 6:**
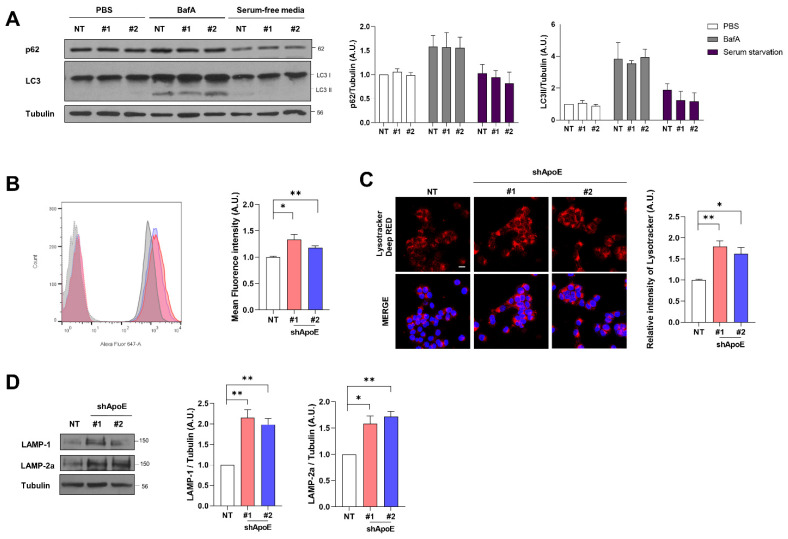
ApoE deficiency enhances chaperone-mediated autophagy in SH-SY5Y cells. (**A**) NT and ApoE KD #1, #2/A53T α-syn-EGFP OE SH-SY5Y cells were treated with 100 nM bafilomycin A1 for 24 h or cultured in serum-free media for 24 h. The cells were then lysed with RIPA buffer and samples were analyzed by Western blot. Values were derived from three independent experiments (*n* = 3). (**B**) The cells were incubated with 50 nM lysotracker for 30 min and then analyzed by flow cytometry. Dotted lines indicate values from samples without lysotracker treatment. Values were derived from three independent experiments (*n* = 3). (**C**) The cells were treated with 50 nM lysotracker for 30 min and their intensity was analyzed by confocal microscopy. Values were derived from three independent experiments (*n* = 3). (**D**) The cells were lysed with RIPA buffer and analyzed by Western blot. Values were derived from three independent experiments (*n* = 3). * *p* < 0.05, ** *p* < 0.01 against the control; one-way ANOVA. Blue indicates DAPI staining. Scale bar indicates 20 μm.

The attenuation of α-syn release can induce the intracellular accumulation of α-syn in neurons. However, endogenous α-syn and exogenously overexpressed A53T-EGFP α-syn did not accumulate intracellularly (Figure S1B,D). In addition, immunocytochemistry analysis showed no difference in A53T α-syn-EGFP aggregates between control and ApoE KD cells (Figure 4B). The α-syn aggregates are degraded mainly by the autophagy-lysosomal system [46,47,48], suggesting that the reduced release from cells may be due to increased autophagic activity. We monitored autophagic activity in both cell types. The levels of LC3II and p62 in ApoE KD cells were similar to those in the control cells, even in response to bafilomycin and serum starvation to block and promote autophagy, respectively [49]. This suggests that autophagic flux was not changed by ApoE deficiency (Figure 6A). However, when we evaluated lysosomal contents using LysoTracker, we found that lysosomal contents were increased in ApoE KD cells (Figure 6B,C). The level of LAMP-1, a glycoprotein abundantly expressed on the lysosomal membrane and a commonly used marker of lysosomal amounts, was also increased, as was that of LAMP-2a, a receptor for chaperone-mediated autophagy (CMA) substrates [50] (Figure 6D). This suggests that ApoE regulates α-syn homeostasis via the autophagy-lysosomal system, even though the autophagic flux was not affected.

### 2.7. The Propagation of α-Syn Is Reduced in ApoE KO Mice

**Figure 7:**
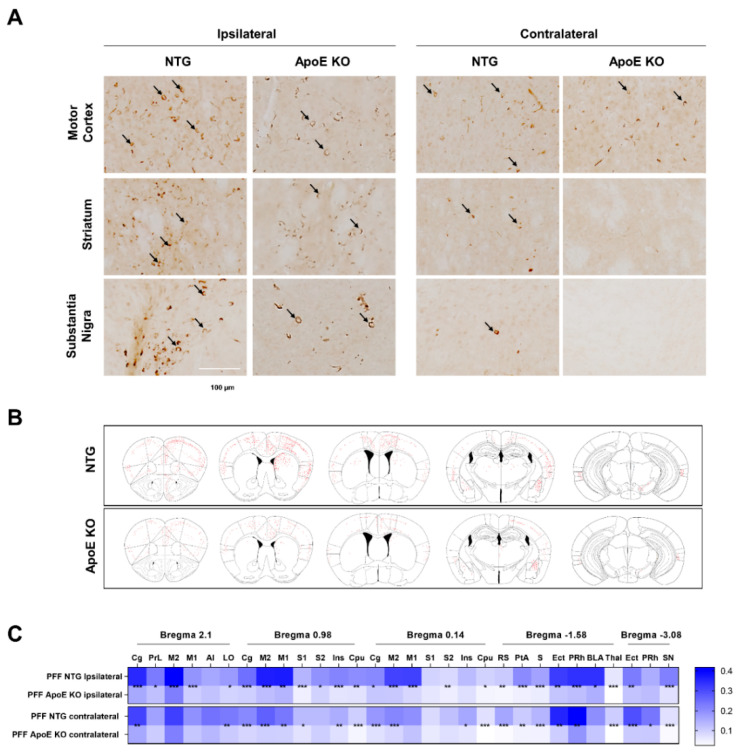
The propagation of α-syn is reduced in ApoE KO mice. (**A**) The 2–3-month-old WT (*n* = 13) and ApoE KO (*n* = 9) mice were injected into the striatum with 10 μg of recombinant mouse α-syn fibrils. After 12 weeks, immunohistochemistry of pSer129 α-syn was performed. Arrow indicates pSer129 α-syn-positive inclusions. Scale bar indicates 100 μm. (**B**) Schematic representation of α-syn inclusion pathology detected by pSer129 α-syn staining. (**C**) Heatmap shows the percentage of thresholds area detected with pSer129 α-syn pathology. * *p* < 0.05, ** *p* < 0.01, *** *p* < 0.001 against the control by unpaired *t*-test.

The stereotaxic injection of preformed mouse α-syn fibrils has been reported to accelerate the formation of intracellular LB- and LN-like inclusions in young wild-type (WT) mice, which represents a useful animal model for the elucidation of the prion-like propagation of α-syn [51]. We injected mouse α-syn fibrils into the unilateral striatum of 8-week-old WT and ApoE KO mice. As shown in Figure 7, Figures S2 and S3, α-syn lesions were detected in the brain by immunohistochemistry of pSer129 α-syn, a marker of pathological α-syn [52] at 90 days post-injection, which was consistent with a previous study [51]. However, the propagation of α-syn lesions was diminished in the brains of ApoE KO mice compared to WT mice (Figure 7), suggesting that ApoE regulates the propagation of α-syn in vivo.

## 3. Discussion

ApoE is predominantly expressed in astrocytes and other cell types in the CNS, including neurons and microglia [27,53]. Gathering evidence suggests that ApoE derived from different cells within the CNS has distinct physiological and pathophysiological functions [27,54,55,56]. The ApoE expressed in microglia is involved in switching between homeostatic and disease-associated phenotypes [57]. ApoE3 KI neurons show increased neuronal activity compared to ApoE4 KI neurons [58]. The overexpression of human ApoE4 in neurons results in the hyperphosphorylation of the microtubule-associated protein tau [54]. A recent study demonstrated that the neuronal expression of ApoE correlates strongly with immune response pathways in neurons [59], suggesting that microglial or neuronal ApoE, apart from astrocytic ApoE, may also be involved in AD pathogenesis. In the present study, we demonstrate the role of neuronal ApoE in cell-to-cell transmission of α-syn.

We observed that the overexpression of A53T α-syn increased ApoE expression in neurons. In addition, the treatment of SH-SY5Y cells with α-syn fibrils also upregulated ApoE expression, suggesting that neuronal ApoE may be involved in α-syn pathology. Although the polymorphisms of ApoE are known to be associated with the risk of AD development, ApoE expression is also involved in AD [14]. Likewise, it has been reported that ApoE expression increases in the neurons of patients with PD [18,19], which is partly supported by our findings.

The pathological spread of α-syn has gained much attention recently, as its spread is composed of the release from cells, the uptake into other cells, and the intracellular aggregation formation with endogenously expressed α-syn [8,60]. In the current experiments, cell-derived α-syn was taken up into ApoE KD cells to a lesser extent than into the control cells. In contrast, overexpression of ApoE isoforms increased the uptake of α-syn, although the effect did not differ among isoforms. Previous studies have suggested that ApoE isoforms have different functions in various pathologies [56,61]. We cannot completely explain the discrepancy between these results. However, it may be due to the different model systems used. The increased risk of AD is 3–4-fold in heterozygotes and 9–15-fold in ApoE4 homozygotes compared to that in non-carriers of ApoE4 [61]. The SH-SY5Y cells have an ApoE3/E3 background [62]. Accordingly, although the expression level of endogenous ApoE3 is relatively low in SH-SY5Y cells, it may affect the uptake of α-syn in ApoE isoform OE cells, which results in no difference in the effect among isoforms. Furthermore, ApoE regulates cholesterol homeostasis [63]. Hepatocytes [64] and dendritic cells [65] from ApoE KO mice have higher levels of intracellular cholesterol. Cholesterol ester accumulation has also been observed in ApoE-deficient cerebral organoids [66]. Consistent with previous studies, we observed increased intracellular cholesterol levels in ApoE KD SH-SY5Y cells. Cholesterol in the plasma membrane is the main constituent of lipid rafts [67], which suggests that ApoE may regulate lipid raft-dependent endocytosis [68,69] and supports our finding. A recent study demonstrated that cholesterol supplementation reduces the entry of tau aggregates and almost completely blocks seeded aggregation [70], which is also similar to our observation.

Additionally, intracellular cholesterol levels regulate LDLR expression via the SREBP-2/SCAP/Insig-1 signaling pathways [71]. Specifically, LDLR and LRP-1 are expressed in both neurons and glia. However, LDLR is expressed more in glia than in neurons [72], whereas LRP-1 is expressed predominantly in neurons [73]. We observed that LDLR and LRP-1 levels were downregulated in ApoE KD cells. Furthermore, LDLR and LRP-1 have been reported to bind Aβ and facilitate its uptake [74]. These receptors play a role in amyloid precursor protein (APP) processing or clearance of Aβ, thereby affecting the balance between Aβ production and clearance [75]. Likewise, the interaction of α-syn fibrils with the plasma membrane decreased in LDLR and LRP-1 KO SH-SY5Y cells. Cell-derived α-syn was taken up into LDLR and LRP-1 KO SH-SY5Y cells to a lesser extent than into control cells, suggesting that LDLR and LRP-1 may be receptors for α-syn fibrils and that may be responsible for α-syn uptake, which is supported by a previous study demonstrating that LRP-1 seems to be involved in α-syn efflux to the periphery in mice [76]. However, α-syn shares striking structural similarities with ApoE [77] and contains two cholesterol-binding domains [78]. Furthermore, α-syn has a strong propensity to bind to lipid membranes, particularly in regions enriched with cholesterol [79], and it interacts with ApoA1, ApoE, and ApoJ, which are found in the HDL sub-fraction of lipoproteins, in the blood plasma, either directly or indirectly [80]. Moreover, α-syn has also been reported to bind apolipoproteins, including ApoE, ApoA1, and ApoJ, in CSF. Transmission electron microscopic analysis of CSF samples also showed the colocalization of α-syn and ApoE in lipoprotein vesicles [19]. Accordingly, α-syn may be taken up by neuronal cells, together with lipoproteins, by LDLR or LRP-1. Given that ApoE regulates lipid raft-dependent endocytosis, other factors for α-syn uptake, apart from LDLR or LRP-1, may be affected. We currently cannot completely examine these possibilities, and further studies are required to decipher this.

We also found that a low amount of extracellular α-syn was taken up by ApoE KD SH-SY5Y cells, leading to a reduced induction of α-syn aggregation with intracellularly expressed α-syn using a coculture assay. However, interestingly, fewer double fluorescence-labeled aggregation puncta were observed in control cells as well as ApoE KD cells, suggesting that ApoE KD cells may release less α-syn. Further studies showed that less α-syn was released from ApoE KD cells. Previously, we observed that c-Src deficiency in neurons impairs α-syn uptake by regulating endocytosis and α-syn release by regulating autophagy [34]. Accordingly, we explored whether ApoE deficiency affected autophagy. Under our experimental conditions, we observed that autophagic flux was unaffected by ApoE deficiency; instead, the lysosomal content was increased. Additionally, the level of LAMP-2a increased. Given that misfolded α-syn is known to be degraded by CMA [46,47] and that the level of LAMP-2a in the lysosomal membrane directly correlates with CMA activity [50], an increase in LAMP-2a expression may affect CMA and lead to a reduction in the release of α-syn without intracellular α-syn accumulation.

Finally, we observed that α-syn propagation was decreased in ApoE KO mice, which supports previous findings of increased α-syn propagation in ApoE4 KI/A53T TG mice [21,22]. Similarly, a lack of ApoE has been reported to attenuate Aβ deposition in APPsw mice [81], suggesting a common role for ApoE in two different pathologies. Our in vivo findings are limited by the fact that we did not address whether neuronal, glial, or both types of ApoE lead to these effects. Nevertheless, considering our in vitro findings, it seems that neuronal ApoE also plays an important role in α-syn propagation, in addition to astrocytic ApoE. Further studies are needed to clarify the role of neuronal ApoE in α-syn propagation in vivo.

## 4. Materials and Methods

### 4.1. Reagents and Antibodies

Antibody against α-syn (#610787) was purchased from BD Biosciences (Franklin Lakes, NJ, USA). Antibody against LRP-1 (#64099S) was supplied by Cell Signaling Technology (Danvers, MA, USA). Antibodies against LDLR (#ab52818), tubulin (#ab6046), and pSer129 α-syn (#ab51253 for Western blotting) were purchased from Abcam (Cambridge, UK). Antibodies against pSer129 α-syn (#015-25191 for immunohistochemistry) were purchased from Wako (Richmond, VA, USA). Antibodies against EGFP (#B-2), ApoE (#A.14), and GAPDH (#6C5) were from Santa Cruz Biotechnology (Santa Cruz, CA, USA). Antibodies against LC3 (#L8918), retinoic acid (RA) (#R2625), and bafilomycin A1 (#B1793) were obtained from Sigma-Aldrich (St. Louis, MO, USA). Rhodamine-conjugated transferrin (#T23365), boron-dipyrromethene (BODIPY) FL C5-lactosylceramide (#D13951), and LysoTracker Deep Red (#L12492) were purchased from Invitrogen (Carlsbad, CA, USA). Monomeric human α-syn was prepared as described previously [82]. The plasmid for mouse α-syn (pD454-SR mouse α-syn, #89075) was obtained from Addgene. Monomeric mouse α-syn was prepared as monomeric human α-syn. To prepare α-syn fibrils, 5 mg/mL monomeric α-syn was incubated at 37 °C with continuous agitation at 2× *g* for 1 week using a Thermomixer F1.5 (Eppendorf, Hamburg, Germany), and stored at −80 °C until use. The α-syn fibrils were sonicated on ice for 1 min, and on–off switched for 1 s at 100 W using an ultrasonic processor VC 505 (Sonics & Materials, Inc., Newtown, CT, USA) before use. The status of the α-syn fibrils was determined by thioflavin T-binding assays and electron microscopy (Figure S4).

### 4.2. Plasmids and Generation of Stable Cell Lines

Plasmids for ApoE2, ApoE3, and ApoE4 were provided by Bradley Hyman (Addgene plasmids #87085, #87086, and #87087, respectively) [83]. All plasmids were confirmed by sequencing. The ApoE KD SH-SY5Y cells were prepared using lentiviral constructs expressing shRNA against human ApoE (KD #1: TRCN10913, KD #2: TRCN371332) (Sigma-Aldrich). Lentiviruses were generated by transfecting HEK293TN cells with a mixture of pLKO.1-puro containing shRNAs against ApoE, pGAG-pol, and pVSV-G using Lipofectamine 2000 (Invitrogen). Supernatants containing the lentiviruses were collected 48 h after transfection. Samples were centrifuged at 250× *g* for 5 min, filtered with a 0.45 μm syringe filter, and added to SH-SY5Y cells. The stable cell lines were selected using puromycin. The A53T α-syn-EGFP and A53T α-syn-mCherry OE SH-SY5Y cells were prepared as described previously [33]. Next, ApoE KD/A53T α-syn-EGFP OE SH-SY5Y cells were generated using lentiviral transfection of A53T α-syn-EGFP in ApoE KD SH-SY5Y cells and selected using a FACSAria III. The lentiCRISPR system was used with a recombinant lentiCRISPRv2 vector, as described previously [84], to generate stable cell lines of LDLR KO and LRP-1 KO SH-SY5Y cells. Briefly, the lentiCRISPRv2 vector (Addgene, #52961) was cut with BsmBI and ligated with human LDLR and LRP-1 annealed oligonucleotides designed by CHOPCHOP (LDLR:5′-TGACATCGGAACCTGTGAGG-3′, LRP-1:5′-ATGAGGGGGCATACCAGAGG-3′). Viral supernatants from transfected HEK293T cells were used to establish stable cell lines. Stable cell lines were selected using puromycin, and further selected from single colonies.

### 4.3. Cell Culture

The SH-SY5Y cells were grown in Dulbecco’s Modified Eagle’s Medium (Hyclone, Logan, Utah) with 10% fetal bovine serum (GenDEPOT, Katy, TX, USA) and maintained at 37 °C in a humidified atmosphere of 5% CO_2_ and 95% air. Primary cortical neurons were prepared from postnatal day 1 C57BL6/J mice (DBL, Eumseong, Korea) and maintained in neurobasal medium (Invitrogen) with GlutaMAX™-I (Gibco, Grand Island, NY, USA) and B-27 supplement (Invitrogen).

### 4.4. Western Blot

Cells were lysed in ice-cold RIPA buffer (25 mM Tris-HCl, pH 7.4, 150 mM NaCl, 1% sodium deoxycholate, 1% Triton X-100, and 0.1% SDS) and a protease inhibitor mixture (Calbiochem, San Diego, CA, USA) for 20 min on ice after sonication for 3 s. Lysates were cleared by centrifugation at 13,000× *g* for 30 min at 4 °C. The supernatants were collected, mixed with sample buffer, resolved by SDS-PAGE, transferred to a nitrocellulose membrane, and immunoblotted with the indicated antibodies. They were then detected using an enhanced chemiluminescence system (West Save Gold, AbFrontier, Seoul, Korea).

### 4.5. Confocal Microscopy

Cells cultured on coverslips were washed twice with PBS and fixed in 4% paraformaldehyde for 10 min at room temperature; the fixed cells were then washed with PBS and incubated with PBS containing 0.1% Triton X-100 for 10 min at room temperature. After washing with PBS, the cells were blocked with PBS containing 1% bovine serum albumin (GenDEPOT) for 1 h at room temperature and then incubated overnight with primary antibodies at 4 °C. Preparations were then stained with fluorescence-conjugated secondary antibody (Jackson Immunoresearch, West Grove, PA, USA) for 1 h, mounted, and observed using an LSM710 confocal microscope (Carl Zeiss, Jena, Germany) or LAS X (Leica, Wetzlar, Germany) at the Three-Dimensional Immune System Imaging Core Facility of Ajou University.

### 4.6. Dual Chamber and Coculture Assays

The A53T α-syn-EGFP OE SH-SY5Y cells were used as donor cells and differentiated by treatment with 50 μM RA for 5 days. The SH-SY5Y cells or primary cortical neurons in a 12-well plate as recipient cells were cocultured with differentiated A53T α-syn-EGFP OE SH-SY5Y cells cultured on the insert for 24 h. Recipient cells were prepared for staining. To measure the amount of internalized α-syn, five random fields were selected, and the intensities of more than 100 cells were analyzed using MetaMorph software (Molecular Devices, San Jose, CA, USA). For the coculture assay, A53T α-syn-EGFP OE SH-SY5Y cells and A53T α-syn-mCherry OE SH-SY5Y cells were cocultured on a coverslip in a 12-well plate (1:1 ratio) for 5 days in the presence of 50 μM RA. The cells were then prepared for confocal microscopy. Five random fields were selected, and more than 100 cells were analyzed. Cells containing double fluorescence-labeled puncta were counted manually. The number of A53T α-syn-EGFP OE SH-SY5Y cells containing double fluorescence-labeled puncta and the number of A53T α-syn-mCherry OE SH-SY5Y cells containing double fluorescence-labeled puncta were counted separately and expressed as percentages of the total cells analyzed.

### 4.7. Cholesterol Measurement

For filipin3 staining, cells cultured on coverslips were washed twice with PBS and fixed in 4% paraformaldehyde for 60 min at room temperature; the fixed cells were washed twice with PBS, incubated with 1.5 mg/mL glycine in PBS for 10 min at room temperature, and then stained with 50 μg/mL of filipin3 in PBS for 2 h at room temperature. The samples were washed twice with PBS, mounted without DAPI, and observed using LAS X. Intracellular cholesterol was measured using the EZ-Total Cholesterol Assay kit (#DG-TSC100, Seoul, Korea) according to the manufacturer’s instructions. Briefly, the cells were washed twice with PBS and scraped for homogenization in a buffer (isopropanol: chloroform: NP40, 7:2:1). The samples were centrifugated at 13,000× *g* for 10 min. The supernatants were collected and dried until they had disappeared and the lipid pellets were dissolved. Cholesterol levels were measured.

### 4.8. Preparation of Conditioned Media from SH-SY5Y Cells

The SH-SY5Y cells were differentiated by treatment with 50 μM RA for 5 days. After differentiation, the medium was replaced with serum-free DMEM and the cells were cultured for 1 d. The media were collected and centrifuged at 1800× *g* for 10 min to remove debris. Supernatants were collected and 50-fold concentrated using vivaspin 2 (SARTORIUS, Göttingen, Germany), according to the manufacturer’s instructions. More detailed, 5 mL of supernatants were placed in vivaspin 2(10,000 MWCO PES) and centrifuged at 3000× *g* for 20 min. After centrifugation, 100 μl of concentrated media were pipetted. Concentrated media were transferred into a 1.7 mL e-tube and stored at −80 °C until use. 

### 4.9. Measurement of α-Syn in Culture Media

Fifty-fold concentrated culture media were used as the samples. ELISA was performed using the Human SNCA (synuclein alpha) ELISA kit (#E-EL-H0983, Elabscience, Houston, TX, USA), according to the manufacturer’s instructions.

### 4.10. α-Syn Real-Time Quaking-Induced Conversion (RT-QUIC) Assay

For α-syn RT-QUIC, the working solution comprised 40 mM phosphate buffer (pH 8.0), 170 mM NaCl, 0.00125% SDS, 10 μM thioflavin T, and 0.1 mg/mL monomeric α-syn. Each well of a black 96-well plate with a clear flat-bottom 96-well plate (#3631, CORNING, Kennebunk, ME, USA) contained 5 μL samples, 95 μL working solution, and 20 mg of 0.5 mm beads (#NC0450473, Bartlesville, OK, USA) for a final reaction volume of 100 μL. Next, the plate was sealed with a plate sealer (Nalgene Nunc International, Rochester, NY, USA), and reactions were initiated in Synergy H1 (Biotek, Winooski, VT, USA) with alternating 1 min shaking and rest cycles (0.034× *g*) at 37 °C. Thioflavin T fluorescence readings were recorded at excitation and emission wavelengths of 452 and 488 nm, respectively, every 15 min for 50 h.

### 4.11. Animals and Intracerebral Injection

ApoE KO mice (B6.129P2-Apoetm1Unc/J) were obtained from the Jackson Laboratory (Farmington, CT, USA), and C57BL/6J mice were obtained from Orient Bio (Sungnam, Korea). Two- to three-month-old C57BL/6J and ApoE KO mice were anesthetized with an intraperitoneal injection of 2,2,2-tribromoethanol (250 mg/kg) and injected into the striatum with sterile PBS or 10 μg of recombinant mouse WT α-syn fibrils (5 mg/mL) (coordinates: anterior–posterior, +1.0 mm; medial–lateral, −1.8 mm; dorsal–ventral, −3.2 mm from the bregma) using a 10 μL Hamilton syringe with a single needle (33 gauge) at an injection rate of 0.4 μL/min; the needle was placed at the injection site for 10 min. All animal procedures were conducted in accordance with the guidelines established by the Ethics Review Committee of Ajou University School of Medicine (IACUC No. 2019-0044).

### 4.12. Immunohistochemistry

The brain was removed after perfusion with PBS, fixed in 4% paraformaldehyde for 24 h, and cryoprotected in 30% sucrose in PBS for 4 days. Fixed brains were cut on a vibratome (Leica, Wetzlar, Germany) at a thickness of 35 μm. The free-floating sections were washed three times for 10 min in PBS and treated with 3% H_2_O_2_ in PBS for 5 min to inactivate endogenous peroxidase, followed by blocking with 1% BSA/0.2% Triton X-100 in PBS for 1 h. Sections were incubated with pSer129 α-syn antibody overnight at 4 °C. After incubation with the biotinylated secondary antibody (Vector Laboratories Burlingame, CA) for 1 h at room temperature, sections were washed with PBS and incubated with ABC reagent (Vector Laboratories Burlingame, CA, USA) for 1 h. Sections were then stained using 3′3′-diaminobenzidine (DAB) (Sigma) at room temperature, mounted on slides, dehydrated with ethanol and xylene, mounted with Permount solution, coverslipped, and analyzed using BX51 (Olympus, Tokyo, Japan) and Picture Frame software (MBF Bioscience Inc., Williston, VT, USA).

### 4.13. Quantitative pSer129 α-Syn Pathology

We quantified pSer129 α-syn pathology as described in a previous study [85] with slight modifications. We selected reference coronal section images, relative to bregma: 2.10 mm, 0.98 mm, 0.14 mm, −1.58 mm, and −3.08 mm [86]. The reference images were imported into MetaMorph software to allow annotation and quantification of the percentage area occupied by pSer129 α-syn pathology. Standardized annotations were drawn to allow independent quantification of 30 regions and modified to match each brain region. After annotation, the minimum threshold was applied to the brain to ensure that no nonpathological signals were detected. Regional measurements were used for all stained sections, and data analysis measures for each region were recorded.

### 4.14. Statistical Analysis

All values are expressed as mean ± SEM. Statistical significance was evaluated using GraphPad Prism software (San Diego, CA, USA).

## 5. Conclusions

We demonstrated that neuronal ApoE regulates α-syn uptake via LRP-1 and LDLR expression and α-syn release via CMA activity, thereby contributing to α-syn propagation. In addition, we found that α-syn propagation is attenuated in ApoE-KO mice. This study will help our understanding of the molecular mechanisms underlying α-syn propagation.

## Data Availability

Not applicable.

## Supplementary Materials

The supporting information can be downloaded at: https://www.mdpi.com/article/10.3390/ijms23158311/s1.
