# Supplementary material for: Neuronal ApoE Regulates the Cell-to-Cell Transmission of α-Synuclein
Source: Int J Mol Sci. 2022 Jul 27;23(15):8311. doi: 10.3390/ijms23158311 (PMC9369063; doi:10.3390/ijms23158311)
Supplement: Supplementary file 1 [file ijms-23-08311-s001.zip › ijms-1802070-supplementary.pdf]

Supplementary Materials

Neuronal ApoE regulates the cell-to-cell transmission of α-synuclein

Seo-Jun Kang[1,2,3], Soo-Jeong Kim[2], Hye Rin Noh[1,2,3], Beom Jin Kim[1,2,3], Jae-Bong Kim[1,2,3], Uram Jin[1,2,3,4], Sun Ah Park[2,3,5,6] and Sang Myun Park[1,2,3,*]

[1]Department of Pharmacology, Ajou University School of Medicine, Suwon, Korea, [2]Center for Convergence Research of Neurological Disorders, Ajou University School of Medicine, Suwon, Korea, [3]Neuroscience Graduate Program, Department of Biomedical Sciences, Ajou University School of Medicine, Suwon, Korea, [4]Department of Cardiology, Ajou University School of Medicine, Suwon, Korea, [5]Department of Anatomy, Ajou University School of Medicine, Suwon, Korea, [6]Department of Neurology, Ajou University School of Medicine, Suwon, Korea

* Correspondence: Dr. Sang Myun Park, Department of Pharmacology, Ajou University School of Medicine, 164, Worldcup-ro, Yeongtong-gu, Suwon, 16499, Korea, Tel: 82-31-219-5063, Fax: 82-31-219-5069, E-mail: sangmyun@ajou.ac.kr

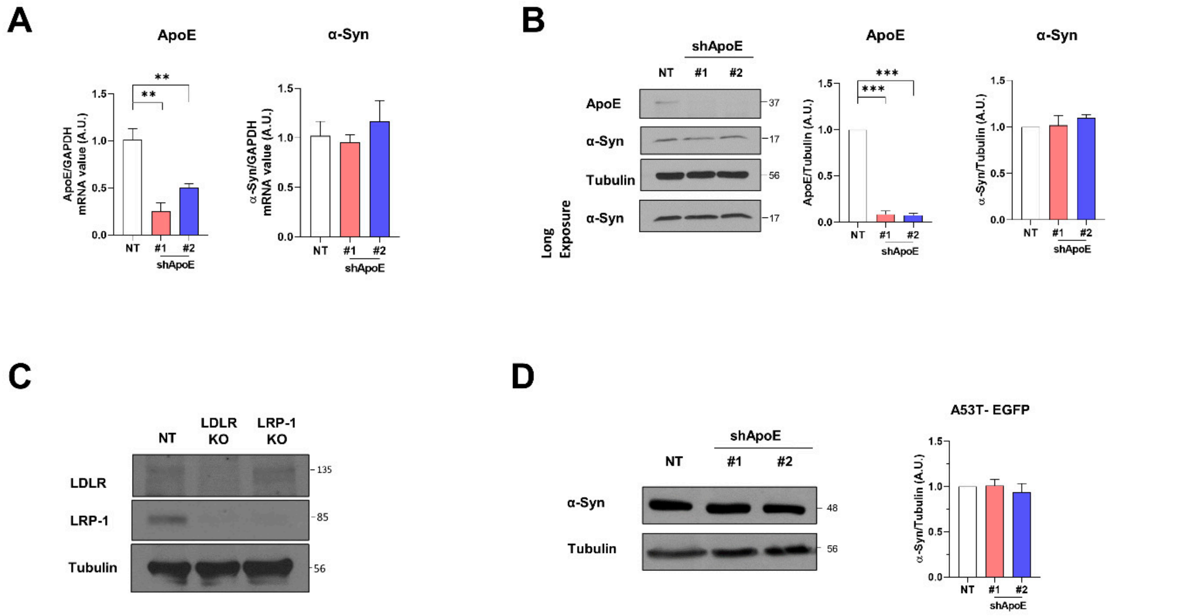

Figure S1. (A) NT, ApoE KD #1, and #2 SH-SY5Y cells were analyzed by RT-PCR. (B) NT, ApoE KD #1 and #2 SH-SY5Y cells were lysed, and western blot was performed with the indicated antibodies. (C) LDLR KO and LRP-1 KO SH-SY5Y cells were lysed, and western blot was performed with the indicated antibodies. Note that the level of LRP-1 in LDLR KO SH-SY5Y cells was also decreased. It may be due that the possible off-target loci for gRNA of LDLR used in the present study are distributed at LRP-1 sequences which are located at 4475 to 4495 bp. (D) NT and ApoE KD #1, #2/A53T α-syn-EGFP OE SH-SY5Y cells were lysed, and western blot was performed with the indicated antibodies. Values were derived from three independent experiments (n = 3). **P < 0.01, ***P < 0.001 against control; one-way ANOVA.

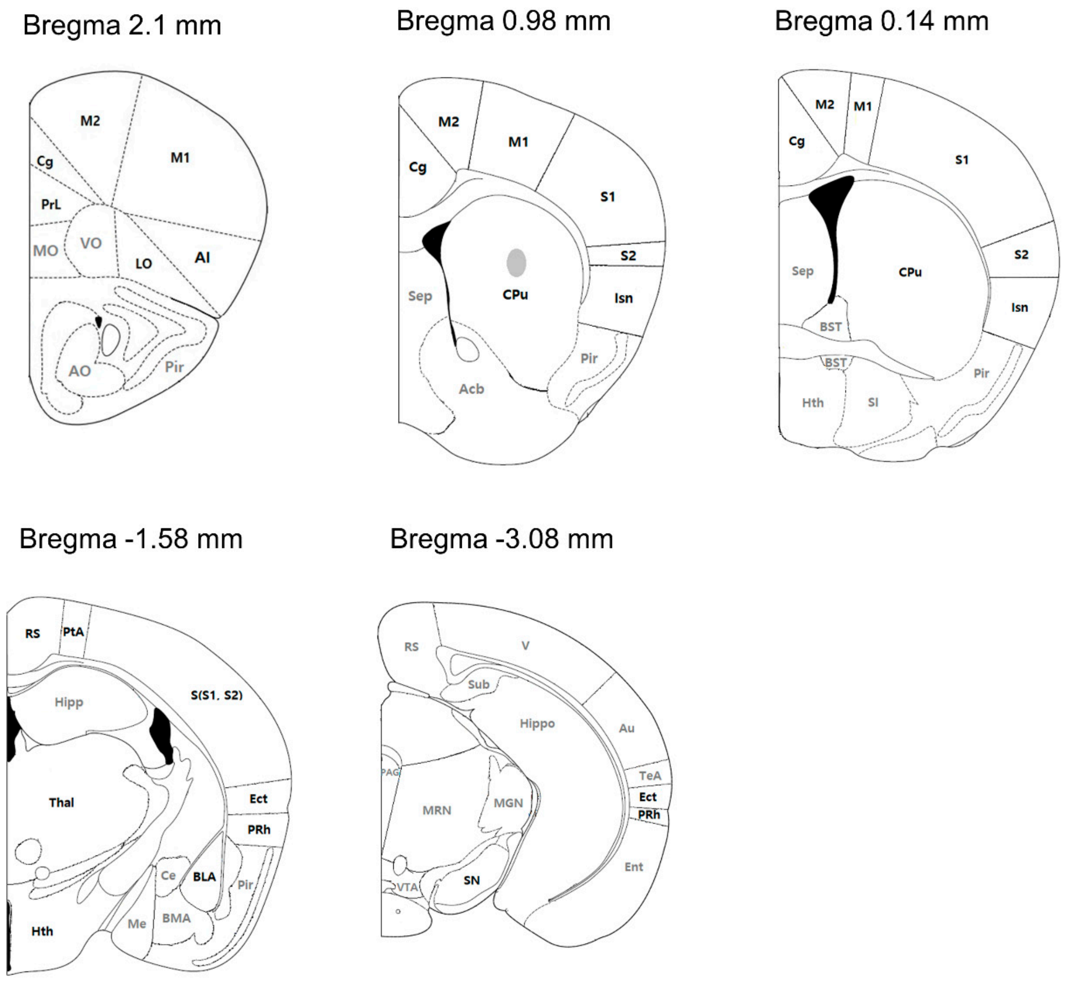

Figure S2. Schematic images of the regions annotated for pathology measures and used in subsequent analysis. Bolded regions were analyzed. Abbreviations : Cg1 = cingulate cortex 1; M1 = motor cortex 1; M2 = motor cortex 2; AI = agranular insular cortex; LO = lateral orbital cortex; Prl = prelimbic cortex; Cg = cingulate cortex; S1 = somatosensory cortex 1; S2 = somatosensory cortex 2; Ins = insular cortex; Cpu = caudate putamen (striatum); RS = retrosplenial cortex; PtA = parietal association cortex; S = somatosensory cortex; Ect = ectorhinal cortex; PRh = perirhinal cortex; CeA = central amygdaloid nucleus; BLA = basolateral amygdaloid nucleus; BMA = basomedial amygdaloid nucleus; Thal = thalamic nuclei; SN = substantia nigra.

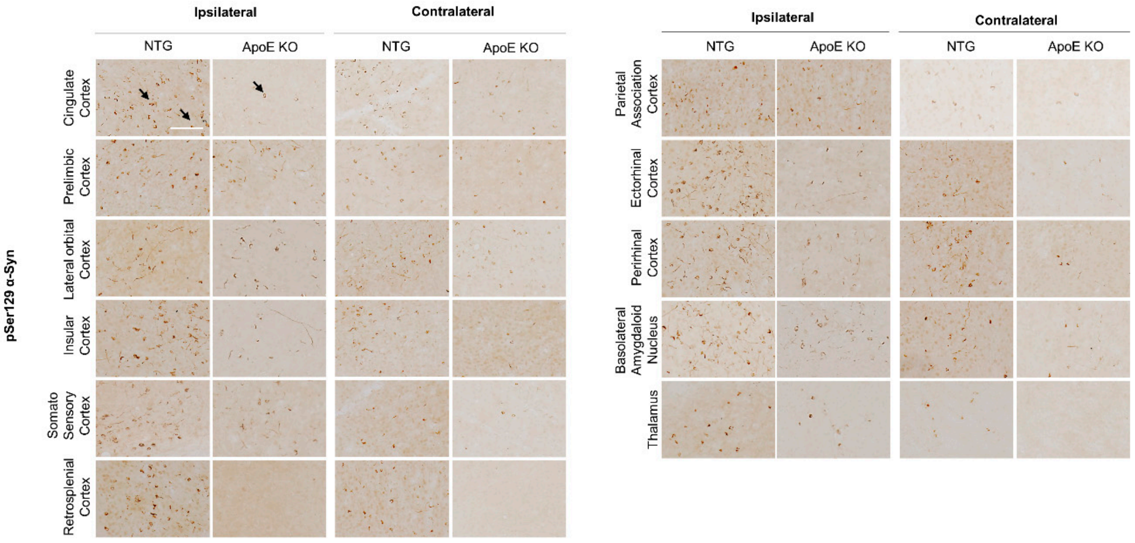

Figure S3. 2–3-month-old WT (n = 13) and ApoE KO (n = 9) mice were injected into the striatum with 10 μg of recombinant mouse α-syn fibrils. After 12 weeks, immunohistochemistry of pSer129 α-syn was performed. Images are representatives of each region. The scale bar indicates 100 μm.

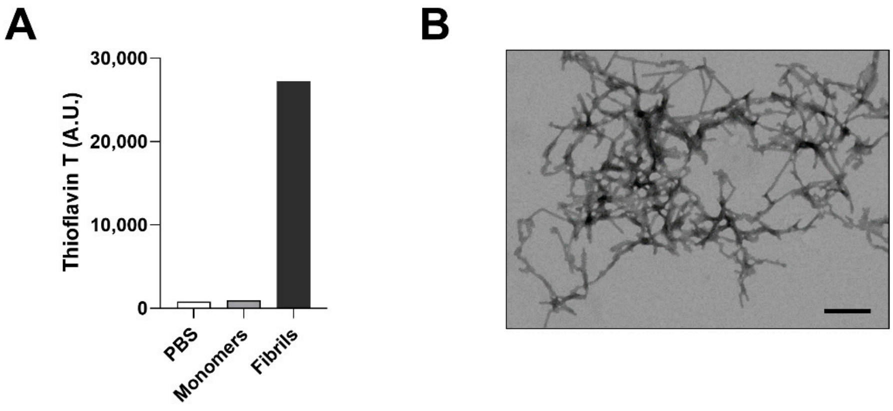

Figure S4. Recombinant α-Syn were analyzed by Thioflavin T assay and electron microscopy. (A) α-Syn monomers and fibrils were mixed with 20 μM thioflavin T assay buffer (250 mM glycine, pH 8.5). The fluorescences were measured at 482 nm with an excitation at 446 nm using Synergy H1 (Biotek, Winooski, VT, USA). (B) α-Syn fibrils were treated onto a carbon-coated copper grid (#CF200-CU, Electron Microscopy Sciences, Ft. Washington, PA, USA) followed by negative staining with 2% uranyl acetate for 2 min. The grid was washed gently with distilled water and dried for 10 mins. α-Syn fibrils were then observed with a SIGMA500 electron microscope (Carl Zeiss, Oberkochen, Germany). The scale bar indicates 200 nm.
